# High‐precision GAFCHROMIC EBT film‐based absolute clinical dosimetry using a standard flatbed scanner without the use of a scanner non‐uniformity correction

**DOI:** 10.1120/jacmp.v11i2.3112

**Published:** 2010-04-17

**Authors:** Heeteak Chung, Bart Lynch, Sanjiv Samant

**Affiliations:** ^1^ Departments of Nuclear and Radiological Engineering University of Florida Gainesville Florida USA; ^2^ Radiation Oncology University of Florida Gainesville Florida USA

**Keywords:** GAFCHROMIC EBT film, absolute dosimetry, flatbed scanner

## Abstract

To report a study of the use of GAFCHROMIC EBT radiochromic film (RCF) digitized with a commercially available flatbed document scanner for accurate and reliable all‐purpose two‐dimensional (2D) absolute dosimetry within a clinical environment. We used a simplified methodology that yields high‐precision dosimetry measurements without significant postirradiation correction. The Epson Expression 1680 Professional scanner and the Epson Expression 10000XL scanner were used to digitize the films. Both scanners were retrofitted with light‐diffusing glass to minimize the effects of Newton rings. Known doses were delivered to calibration films. Flat and wedge fields were irradiated with variable depth of solid water and 5 cm back scatter solid water. No particular scanner nonuniformity effect corrections or significant post‐scan image processing were carried out. The profiles were compared with CC04 ionization chamber profiles. The depth dose distribution was measured at a source‐to‐surface distance (SSD) of 100 cm with a field size of $10\times 10\;{\text{cm}}^{2}$. Additionally, 22 IMRT fields were measured and evaluated using gamma index analysis. The overall accuracy of RCF with respect to CC04 was found to be 2%–4%. The overall accuracy of RCF was determined using the absolute mean of difference for all flat and wedge field profiles. For clinical IMRT fields, both scanners showed an overall gamma index passing rate greater than 90%. This work demonstrated that EBT films, in conjunction with a commercially available flatbed scanner, can be used as an accurate and precise absolute dosimeter. Both scanners showed that no significant scanner nonuniformity correction is necessary for accurate absolute dosimetry using the EBT films for field sizes smaller than or equal to $15\times 15\;{\text{cm}}^{2}$.

PACS number: 87.53.Bn

## I. INTRODUCTION

Film has been used to record ionizing radiation since the original discovery of X‐rays and has remained an important technique through the present time. Since then, film dosimetry has been proven to be a highly reliable and valuable tool for radiation dosimetry. The high spatial resolution of film makes it a useful dosimeter in quality assurance (QA) and research. With the introduction of intensity modulated radiation therapy (IMRT), film dosimetry has found another application for evaluating the high gradient dose distributions generated by IMRT.

Currently, 2 types of film are available for radiation therapy applications: radiographic and radiochromic (solid state polymer based^(1)^) films. Radiographic film (e.g., Kodak XV‐2, Kodak EDR2) has been extensively used for QA, research, and commissioning purposes.^(2)^ ^,^ ^(3)^ However, it has some notable limitations. One of the biggest limitations is that radiographic film contains an emulsion layer that has a high effective atomic number (Z), which causes it to over respond to low energy spectral component.^(4)^ ^,^ ^(5)^ In addition to over response, radiographic film requires a developer and a darkroom, and development can influence the proper development of the film.^(6)^ ^,^ ^(7)^ Because of its immediate archival/retrieval and input capabilities for various clinical software packages, digital imaging is being used with increasing frequency in the clinical setting, decreasing the clinical utilization of radiographic film.

GAFCHROMIC EBT (International Specialty Products, Wayne, NJ) radiochromic film (RCF) is a commercially available film designed to measure the absorbed dose from photon beams with a recommended dose range of 1 cGy to 800 cGy. RCF is composed of radiation‐sensitive dye that is organized into microcrystals and embedded in a gelatin binder. Upon irradiation, a solid state polymerization^(1)^ takes place and the film becomes progressively blue in color. The advantages of RCF over radiographic films are that it is mostly insensitive to the visible light spectrum, it does not require a developer and dark room, and it is easy to handle. RCF is, for the most part, not sensitive to the visible light spectrum although it is more sensitive to short wavelength light than to long wavelength light. According to the manufacturer, a continuous exposure to interior light (such as that produced by cool, white fluorescent bulbs) for 24 hrs results in an optical density (OD) change of ~0.007, which is equivalent to a radiation exposure of up to ~3cGy. Thus, RCF can be handled in room light for several hours before requiring storage. In spite of these technical benefits, RCF has some inherent systematic errors including nonuniformity (although EBT RCF has been significantly improved over previous RCFs, such as MD‐55 and HS, in this regard), temperature and humidity dependence of polymerization, and scanning artifacts.^(1)^ ^,^ ^(8)^

Proper usage of a flatbed scanner is important to minimize significant scanning artifacts. Lynch et al.^(9)^ identified three significant artifacts that can severely limit the accuracy of RCF readout on flatbed scanners. The first effect involves scanner nonuniformity (i.e. bowing effect), by which the variation can be as much as 17% for film profiles in the direction of the CCD array. Others^(^ ^10^ ^–^ ^13^ ^)^ have attempted to correct the scanner nonuniformity effect and were able to improve it by 1.1% to 3.6%. A sophisticated correction matrix for each film batch must be generated that will compensate for the light scattering from the scanner lamp, which depends on pixel location and OD, to obtain such close agreement. Briefly, a set of uniformly irradiated films are scanned and normalized profiles for each film are established. From these normalized profiles, correction factors are obtained, taking the geometrical position and the optical density into consideration. In effect, a three‐dimensional (3D) correction factor is generated. Although this scanner nonuniformity correction method has produced good results, it must be performed for each batch of film, which is labor intensive and thus impractical in a clinical setting.

The second effect is a film rotation effect, which depends on the orientation of the film on the flatbed scanner bed. Lynch et al.^(9)^ reported a 15% variation in OD over the range of angles for the 0 cGy film, which decreased to ~8% for the 300 cGy film. This variation in OD can be minimized with consistent positioning of the films during irradiation and scanning. The third effect depends on the temperature of the scanner bed while scanning and it can result in a variation in OD of up to 7% for low OD.

In the clinical setting, the correction methods stated above can be cumbersome and impractical. In this paper, we report the use of GAFCHROMIC EBT RCF, in conjunction with two flatbed scanners, to obtain good agreement with dose measurements made using an ionization chamber (for cross‐plane and in‐plane directions for open and wedge fields) and with a planar dose (for IMRT fields) without using a scanner nonuniformity correction method. The two flatbed scanners were the Epson Expression 1680 Professional scanner (Epson America Inc., Long Beach, CA) and the Epson Expression 10000XL scanner. For comparison, CC04 (IBA Dosimetry, Schwarzenbruck, Germany) ionization chamber profiles were used as a reference.

To evaluate the IMRT dose distribution, we used a planar dose distribution from the Pinnacle^3^ treatment planning system (Philips Medical Systems, Andover, MA).

## II. MATERIALS AND METHODS

For this study, all irradiations were performed with a 6 MV photon beam from an Elekta Synergy linear accelerator (Elekta Oncology Systems, Crawley, UK). Absolute dose calibration of the linear accelerator was done according to the TG‐51^(14)^ protocol. Calibration conditions were 0.78 cGy/MU at 10 cm depth, a source‐to‐surface distance (SSD) of 90 cm, and a field size of $10\times 10\;{\text{cm}}^{2}$ defined at a source‐to‐axis distance (SAD) of 100 cm. Beam profiles were scanned using a 3D water tank system with a CC04 ionization chamber that has a sensitive volume of $0.04\;{\text{cm}}^{3}$. Water tank profile scans were used as the reference for all open and wedge field profiles. For all CC04 scans, a constant SSD of 90 cm was maintained. For flat fields, three field sizes ($5\times 5\;{\text{cm}}^{2},\;10\times 10\;{\text{cm}}^{2}$, and $20\times 20\;{\text{cm}}^{2}$) and four depths (5 cm, 10 cm, 20 cm, and 25 cm) were used. For wedge fields, two field sizes ($6\times 6\;{\text{cm}}^{2}$ and $10\times 10\;{\text{cm}}^{2}$) and three depths (10 cm, 15 cm, and 25 cm) were used. A depth dose distribution was measured using the CC04 ionization chamber at a SSD of 100 cm with a field size of $10\times 10\;{\text{cm}}^{2}$. The pixel resolution for all profiles and depth dose distributions was maintained at 1 mm. To evaluate the IMRT 2D dose distribution, planar dose from the Pinnacle3 treatment planning system for comparison was used. For IMRT fields, the gantry and collimator angles were maintained at 0 degrees with a SSD of 90 cm, depth of 10 cm, and 5 cm of backscatter material. The planar doses from the treatment planning system and the MapCHECK 2D diode array detector (Sun Nuclear, Melbourne, FL) were also used for the evaluation. A total of 22 IMRT fields from the treatment plans for four patients with head and neck cancers were delivered and analyzed for this study.

### A. Film scanners

Both the Epson Expression 1680 and 10000XL scanners are flatbed color image scanners with xenon gas cold cathode fluorescent lamp and charge‐coupled device (CCD) line sensor. Both systems have transparency units, which were used to scan the RCFs. Both scanners were also retrofitted with light diffusing glass which is effective at minimizing Newton ring artifacts.^(8)^ They have the maximum pixel depth of 48 bits (16 bits per color channel). The maximum scan area of models 1680 and 10000XL were $21.6\times 29.7\;{\text{cm}}^{2}$ and 31 × $43.7\;{\text{cm}}^{2}$, respectively. The physical dimensions of the Epson 1680 scanner were 33.2 cm width, 56.2 cm depth, 13.3 cm height, and 8.5 kg weight. The physical dimensions of the Epson 10000XL scanner were 65.6 cm width, 45.8 cm depth, 15.8 cm height, and 13 kg weight.

### B. Calibration and irradiation protocol

Radiochromic films (Lot # 47261‐07I) were used for flat and wedge fields and depth dose measurements. For IMRT studies, RCFs with Lot # 36306‐001I were used. For each measurement set, calibration films were also irradiated to convert OD to absolute dose. In this study, the term absolute dose or dosimetry refers to a technique in which a known dose is defined at a corresponding OD so that OD can be converted to be expressed in terms of absolute dose. The doses of the calibration films were cross‐calibrated with the measurements from an ADCL calibrated ion chamber, following TG51 protocol.^(14)^ Calibration and measurement films were handled together to minimize variations due to temperature and humidity during irradiation and film development.

**Figure 1:**
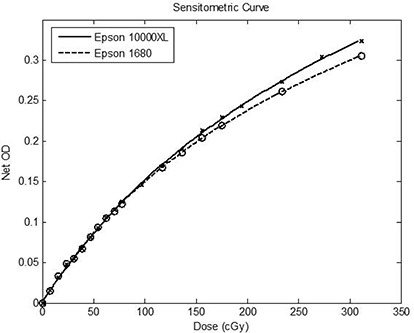
Sensitometric curves for the Epson 1680 (o) and the Epson 10000XL (x) scanners. Two sensitometric curves were generated using the same set of calibration films (Lot # 47261‐071).

Proper film irradiation techniques must be followed to establish an accurate and reproducible sensitometric curve. To accomplish this, blank unirradiated RCF calibration films were cut into $\sim 5\times 5\;{\text{cm}}^{2}$ squares. The films (both the calibration and the measurement films) were marked to maintain consistent orientation during scanning. After these pieces were cut and marked, a reference setup was used to irradiate the films to known doses. The reference setup was a 6 MV isocentric perpendicular setup at a SSD of 90 cm, depth of 10 cm, field size of $10\times 10\;{\text{cm}}^{2}$, and 5 cm of solid water backscatter conditions. In this reference setup, 1 monitor unit (MU) was equal to 0.78 cGy. Once this setup was established, each EBT calibration film was placed just below the 10 cm depth of solid water in the middle of the light field and irradiated to known doses. One piece of calibration film was not irradiated to represent 0 cGy. The calibration dose ranged from 0 cGy to 312 cGy. After the film images were allowed to develop for 24 hrs, RCFs were scanned using the Epson 1680 and Epson 10000XL scanners to establish a net OD versus dose sensitometric curve (Fig. 1). To show the sensitometric curve variability with respect to scanners in Fig. 1, the same set of calibration films were scanned using both scanners. The sensitometric curve was fitted with a fourth‐order polynomial calibration function, which can be used to convert net OD to dose. A calibration function was generated for each scanner.

A series of flat and wedge fields were irradiated with varying field sizes at different depths to obtain cross‐plane and in‐plane profiles. For flat fields, there were three field sizes ($5\times 5\;{\text{cm}}^{2},\;10\times 10\;{\text{cm}}^{2}$, and $20\times 20\;{\text{cm}}^{2}$) with four depths (5 cm, 10 cm, 20 cm, and 25 cm). For wedge fields, there were two field sizes ($6\times 6\;{\text{cm}}^{2}$ and $10\times 10\;{\text{cm}}^{2}$) with three depths (10 cm, 15 cm, and 25 cm). For all flat fields, 200 MU were used for the irradiations while, for all wedge fields, 400 MU were used. For both flat and wedge fields, an SSD of 90 cm was maintained. Except for the $20\times 20\;{\text{cm}}^{2}$ beam, the films were irradiated in an orientation such that the cross‐plane direction was orthogonal to the longer side of the film (portrait mode). Because of the limitation due to film size, the film was positioned so that the cross‐plane of the $20\times 20\;{\text{cm}}^{2}$ beam was parallel to the longer side of the film and the in‐plane profiles were not measured. A depth dose distribution was measured using a CC04 ionization chamber at an SSD of 100 cm with a field size of $10\times 10\;{\text{cm}}^{2}$. For the depth dose setup, RCF was sandwiched between two 10 cm thick solid water blocks. The length (shorter side) of the RCF was aligned with the length of the solid water and laid flat on the couch horizontally. The gantry was rotated 90° and the source‐to‐surface distance was 100 cm with a field size of $10\times 10\;{\text{cm}}^{2}$. For the IMRT plans, the gantry and collimator angles were maintained at 0° with 90 cm, depth of 10 cm, and 5 cm of backscatter material. In all, 22 IMRT fields were studied.

### C. Scanning protocol and analysis

Both scanners were turned on 30 min before scanning to allow them to warm up sufficiently. After the 30 min warm‐up, several scans were made without any films to further warm up both systems. Each calibration and measurement film was then placed individually in the center of the scanning bed, with their orientation maintained constant to minimize film rotation error. Since no scanner nonuniformity correction was applied, it was critical that the film orientation be maintained for each scanner. RCFs were positioned in both scanners in portrait mode (the length of the RCF was orthogonal to the scanning direction). Before each measurement scan, a preview scan was done to verify the film's position. Once verified, the whole scanning area was scanned with 100 dpi resolution (0.254mm/pixel) and saved as a] 48‐bit RGB uncompressed tagged image file format (TIFF) image file.

After the films were imaged and saved in TIFF, a simple code to read and analyze the image data was written using MATLAB version 7.4 software (The MathWorks Inc., Natick, MA). The calibration films were used to generate a fourth‐order polynomial sensitometric curve which was applied to the measurement films to convert the net OD to absolute dose. Only the pixel values from the red channel were used in the analysis. MATLAB was also used to analyze the cross‐plane and in‐plane profiles for the flat and wedge fields. The 2D dose distributions from the film measurements, the planar dose from the treatment planning system, and the 2D diode array detector were used to evaluate the IMRT fields. All three datasets were imported into the 2D diode array detector software for gamma index analysis. The RCF profiles (cross‐plane and in‐plane) and depth dose distributions were resampled to have the same resolution as the CC04 ionization chamber measurements (1 mm per pixel). To evaluate film measurements obtained with the CC04 ionization chamber for a given profile, a central axis (CAX) percent dose difference and the mean of the difference was used. The CAX percent dose difference is the dose difference $\left({\text{CC}04}_{@\text{CAX}}–{\text{RCF}}_{@\text{CAX}}\right)$ at central axis CAX (a single pixel point from the RCF profile) of the profile normalized to the CC04 dose. The mean of the difference is the mean of the dose difference between CC04 and RCF for 50% – 50% (D50) and 80% – 80% (D80) of the profile (Eq. 1). The percentage in D50 and D80 refers to the percent penumbra line (e.g. 50% – 50% refers to the mean of the difference for the points in between 50% of the penumbra region). The mean of the difference would evaluate the agreement between the measurement and the reference for a large number of points, which in turn would provide an overall agreement between the profiles.

(1) $$Mean\_of\_the\_difference=\frac{1}{N}\sum_{i=1}^{N}\left(\frac{CC04_{i}-RCF_{i}}{CC04_{i}}\times 100\right)$$

To evaluate the IMRT fields, the gamma test criteria were 3% dose difference and 3 mm distance‐to‐agreement (DTA). Pass rates of greater than or equal to 90% were considered acceptable since these are the criteria used in our clinic.

## III. RESULTS

### A. Open field profile

**Figure 2:**
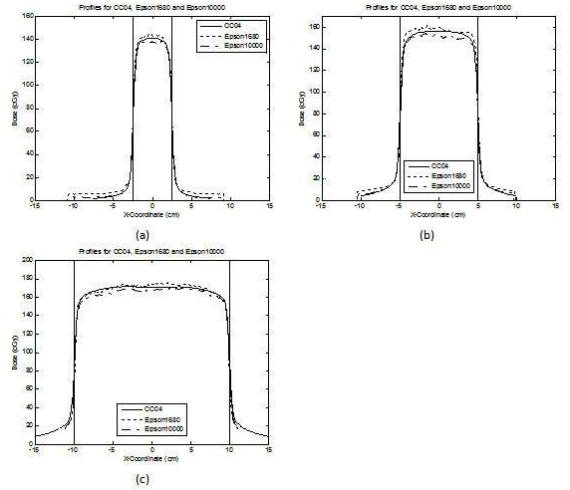
Cross‐plane profiles for flat fields obtained using the scanners and the corresponding CC04 ionization chamber profiles: (a) $5\times 5\;{\text{cm}}^{2}$ field size; (b) $10\times 10\;{\text{cm}}^{2}$ field size; (c) $20\times 20\;{\text{cm}}^{2}$ field size. All three profiles were at a 10 cm depth. The solid vertical lines indicate the 50% penumbra line. The solid line represents the CC04 ionization chamber profile, the dotted line the Epson 1680 profile, and the line‐dot line the Epson 10000XL profile.

Figure 2 shows three cross‐plane profiles for flat fields obtained using the flatbed scanners and the CC04 ionization chamber. All three profiles were at a depth of 10 cm. Figures 2(a), 2(b), and 2(c) correspond to field sizes of $5\times 5\;{\text{cm}}^{2},\;10\times 10\;{\text{cm}}^{2}$, and $20\times 20\;{\text{cm}}^{2}$, respectively. For this dataset, the maximum and minimum CAX percent dose differences were −1.6% and −2.3%, respectively, for the Epson 1680 scanner. For the Epson 10000XL scanner, the maximum and minimum CAX percent doses differences were 2.6% and 1.1%, respectively. The D50 values for the mean of the difference (one standard deviation) for the Epson 1680 scanner were −2.7%(1σ=2.8%),−3.0%(1σ=3.6%), and −0.3%(1σ=3.3%) for field sizes of $5\times 5\;{\text{cm}}^{2}$, $10\times 10\;{\text{cm}}^{2}$, and 20 xby 20 cm^2^, respectively. For the same field sizes, D80 values for the mean of the difference were −2.3%(1σ=2.0%),−2.5%(1σ=2.5%), and −0.6%(1σ=1.9%), respectively. Similarly, for the Epson 10000XL scanner, the D50 values for the mean of the difference were 1.1%(1σ=2.6%),2.2%(1σ=2.2%), and 2.2%(1σ=2.2%) for field sizes of $5\times 5\;{\text{cm}}^{2}$, $10\times 10\;{\text{cm}}^{2}$, and 20 xby 20 cm^2^, respectively. For the same field sizes, D80 values for the mean of the difference were 1.3%(1σ=1.6%),2.2%(1σ=1.4%), and 1.9%(1σ=0.9%), respectively. All other flat field profiles had similar results. Tables 1 and 2 present the results for all of the flat field cross‐plane and in‐plane profiles calculated for D50 and D80.

**Table 1:** Results for flat field cross‐plane and in‐plane profiles for D50 compared with CC04.

	Depth=5cm				Depth=10cm			
	*Epson 1680*		*Epson 10000XL*		*Epson 1680*		*Epson 10000XL*	
*Field Size*	*CAX*	*Mean of Difference*	*CAX*	*Mean of Difference*	*CAX*	*Mean of Difference*	*CAX*	*Mean of Difference*
		*(Standard Deviation)*		*(Standard Deviation)*		*(Standard Deviation)*		*(Standard Deviation)*
$5\times 5\;{\text{cm}}^{2}$	−3.4%	−3.6% (3.4%)	1.3%	0.6% (4.5%)	−1.6%	−2.7% (2.8%)	2.6%	1.1% (2.6%)
$10\times 10\;{\text{cm}}^{2}$	−1.9%	−3.9% (4.5%)	1.6%	0.2% (2.7%)	−2.3%	−3.0% (3.6%)	2.5%	2.2% (2.2%)
$20\times 20\;{\text{cm}}^{2}$	−3.4%	−1.5% (2.8%)	1.5%	1.9% (2.1%)	−2.0%	−0.3% (3.4%)	1.1%	2.2% (2.2%)

	Depth=20cm				Depth=25cm			
	*Epson 1680*		*Epson 10000XL*		*Epson 1680*		*Epson 10000XL*	
*Field Size*	*CAX*	*Mean of Difference*	*CAX*	*Mean of Difference*	*CAX*	*Mean of Difference*	*CAX*	*Mean of Difference*
		*(Standard Deviation)*		*(Standard Deviation)*		*(Standard Deviation)*		*(Standard Deviation)*
$5\times 5\;{\text{cm}}^{2}$	0.7%	0.5% (3.7%)	1.2%	−0.9% (3.2%)	1.3%	1.7% (2.7%)	−2.0%	−1.9% (3.2%)
$10\times 10\;{\text{cm}}^{2}$	−0.8%	−1.6% (3.7%)	−0.4%	−1.2% (2.9%)	2.1%	1.7% (2.7%)	−0.1%	−0.2% (2.0%)
$20\times 20\;{\text{cm}}^{2}$	−0.7%	0.7% (2.4)	−0.2%	0.6% (6.9%)	0.7%	4.3% (13.0%)	−0.6%	3.1% (17.2%)

**Table 2:** Results for flat field cross‐plane and in‐plane profiles for D80 compared with CC04.

	Depth=5cm				Depth=10cm			
	*Epson 1680*		*Epson 10000XL*		*Epson 1680*		*Epson 10000XL*	
*Field Size*	*CAX*	*Mean of Difference*	*CAX*	*Mean of Difference*	*CAX*	*Mean of Difference*	*CAX*	*Mean of Difference*
		*(Standard Deviation)*		*(Standard Deviation)*		*(Standard Deviation)*		*(Standard Deviation)*
$5\times 5\;{\text{cm}}^{2}$	−3.4%	−2.9% (1.6%)	1.3%	0.8% (1.6%)	−1.6%	−2.3% (2.0%)	2.6%	1.3% (1.6%)
$10\times 10\;{\text{cm}}^{2}$	−1.9%	−3.1% (2.4%)	1.6%	0.3% (1.3%)	−2.3%	−2.5% (2.5%)	2.5%	2.2% (1.4%)
$20\times 20\;{\text{cm}}^{2}$	−3.4%	−1.8% (1.6%)	1.5%	1.6% (0.7%)	−2.0%	−0.6% (1.9%)	1.1%	1.9% (0.9%)

	Depth=20cm				Depth=25cm			
	*Epson 1680*		*Epson 10000XL*		*Epson 1680*		*Epson 10000XL*	
*Field Size*	*CAX*	*Mean of Difference*	*CAX*	*Mean of Difference*	*CAX*	*Mean of Difference*	*CAX*	*Mean of Difference*
		*(Standard Deviation)*		*(Standard Deviation)*		*(Standard Deviation)*		*(Standard Deviation)*
$5\times 5\;{\text{cm}}^{2}$	0.7%	0.8% (2.1%)	1.2%	−0.6% (1.8%)	1.3%	2.3% (1.8%)	−2.1%	−1.5% (1.8%)
$10\times 10\;{\text{cm}}^{2}$	−0.8%	−1.0% (2.6%)	−0.4%	−0.9% (1.6%)	2.1%	2.2% (1.7%)	−0.1%	−0.1% (1.6%)
$20\times 20\;{\text{cm}}^{2}$	−0.7%	0.5% (1.5%)	−0.2%	−0.0% (1.1%)	0.7%	2.5% (2.3%)	−0.6%	0.5% (6.7%)

### B. Wedge field

**Figure 3:**
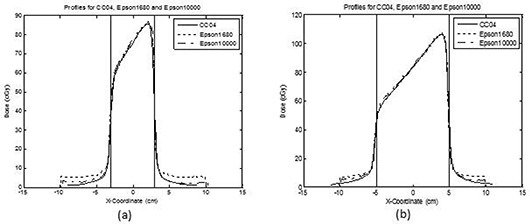
Cross‐plane profiles for wedge fields obtained using the scanners and the corresponding ionization chamber profiles: (a) $6\times 6\;{\text{cm}}^{2}$ field size; (b) $10\times 10\;{\text{cm}}^{2}$ field size. Both profiles were at a 10 cm depth. The solid vertical line indicates the 50% penumbra line. The solid line represents the CC04 ionization chamber profile, the dotted line the Epson 1680 profile, and the line‐dot line the Epson 10000XL profile.

Figure 3 shows the cross‐plane profiles for wedge fields obtained using the scanners and the corresponding CC04 ionization chamber profiles. The profiles correspond to a field size of $6\times 6\;{\text{cm}}^{2}$ (Fig. 3(a) and $10\times 10\;{\text{cm}}^{2}$ (Fig. 3(b) at a depth of 10 cm. For this dataset, the maximum and minimum CAX dose differences were −0.6% and −2.1%, respectively, for the Epson 1680 scanner. For the Epson 10000XL scanner, the maximum and minimum CAX dose differences were −0.4% and −1.2%, respectively. For the Epson 1680 scanner, the D50 values for the mean of the difference were −0.8%(1σ=7.8%) for a field size of $6\times 6\;{\text{cm}}^{2}$ and 0.5%(1σ=5.7%) for a field size of $10\times 10\;{\text{cm}}^{2}$. For the same field sizes, D80 values for the mean of the difference were −1.2% (1σ=2.0%) and −0.1% (1σ=1.7%), respectively. For the Epson 10000XL scanner, the D50 values for the mean of the difference were −2.9%(1σ=5.2%) for a field size of $6\times 6\;{\text{cm}}^{2}$ and −1.3%(1σ=3.1%) for a field size of $10\times 10\;{\text{cm}}^{2}$. The D80 values for the mean of the difference were −2.8% (1σ=2.9%) and −1.3%(1σ=2.8%) for a field size of $6\times 6\;{\text{cm}}^{2}$ and $10\times 10\;{\text{cm}}^{2}$, respectively. All other wedge field profiles had similar results. Tables 3 and 4 present the results for all of the wedge field cross‐plane and in‐plane profiles calculated for D50 and D80.

**Table 3:** Results for wedge fields for D50 compared with CC04.

	Depth=10cm				Depth=15cm				Depth=25cm			
	*Epson 1680*		*Epson 10000XL*		*Epson 1680*		*Epson 10000XL*		*Epson 1680*		*Epson 10000XL*	
*Field Size*	*CAX*	*Mean of Difference*	*CAX*	*Mean of Difference*	*CAX*	*Mean of Difference*	*CAX*	*Mean of Difference*	*CAX*	*Mean of Difference*	*CAX*	*Mean of Difference*
		*(Standard Deviation)*		*(Standard Deviation)*		*(Standard Deviation)*		*(Standard Deviation)*		*(Standard Deviation)*		*(Standard Deviation)*
$6\times 6\;{\text{cm}}^{2}$	−2.1%	−0.8% (7.8%)	−1.2%	−2.9% (5.2%)	1.4%	1.3% (5.2%)	−0.89%	−2.2% (5.2%)	4.6%	2.8% (6.2%)	−1.3%	−2.5% (5.1%)
$10\times 10\;{\text{cm}}^{2}$	−0.6%	−0.5% (5.7%)	−0.4%	−1.3% (3.1%)	2.4%	1.2% (5.6%)	−0.4%	−2.2% (3.8%)	4.0%	3.1% (2.6%)	−0.9%	−1.2% (3.0%)

	Depth=10cm				Depth=15cm				Depth=25cm			
	*Epson 1680*		*Epson 10000XL*		*Epson 1680*		*Epson 10000XL*		*Epson 1680*		*Epson 10000XL*	
*Field Size*	*CAX*	*Mean of Difference*	*CAX*	*Mean of Difference*	*CAX*	*Mean of Difference*	*CAX*	*Mean of Difference*	*CAX*	*Mean of Difference*	*CAX*	*Mean of Difference*
		*(Standard Deviation)*		*(Standard Deviation)*		*(Standard Deviation)*		*(Standard Deviation)*		*(Standard Deviation)*		*(Standard Deviation)*
$6\times 6\;{\text{cm}}^{2}$	−2.1%	−1.2% (2.0%)	−1.2%	−2.8% (2.9%)	1.4%	1.5% (1.9%)	−0.9%	−1.5% (3.1%)	4.6%	4.3% (3.2%)	−1.3%	−2.0% (2.6%)
$10\times 10\;{\text{cm}}^{2}$	−0.6%	−0.1% (1.7%)	−0.4%	−1.3% (2.8%)	2.4%	0.7% (1.6%)	−0.4%	−2.4% (2.3%)	4.0%	3.8% (3.1%)	−0.96%	−1.2% (2.4%)

**Table 4:** Results for wedge fields for D80 compared with CC04.

	Depth=10cm				Depth=15cm				Depth=25cm			
	*Epson 1680*		*Epson 10000XL*		*Epson 1680*		*Epson 10000XL*		*Epson 1680*		*Epson 10000XL*	
*Field Size*	*CAX*	*Mean of Difference*	*CAX*	*Mean of Difference*	*CAX*	*Mean of Difference*	*CAX*	*Mean of Difference*	*CAX*	*Mean of Difference*	*CAX*	*Mean of Difference*
		*(Standard Deviation)*		*(Standard Deviation)*		*(Standard Deviation)*		*(Standard Deviation)*		*(Standard Deviation)*		*(Standard Deviation)*
$6\times 6\;{\text{cm}}^{2}$	−2.1%	−1.2% (2.0%)	−1.2%	−2.8% (2.9%)	1.4%	1.5% (1.9%)	−0.9%	−1.5% (3.1%)	4.6%	4.3% (3.2%)	−1.3%	−2.0% (2.6%)
$10\times 10\;{\text{cm}}^{2}$	−0.6%	−0.1% (1.7%)	−0.4%	−1.3% (2.8%)	2.4%	0.7% (1.6%)	−0.4%	−2.4% (2.3%)	4.0%	3.8% (3.1%)	−0.96%	−1.2% (2.4%)

### C. Depth dose profile

**Figure 4:**
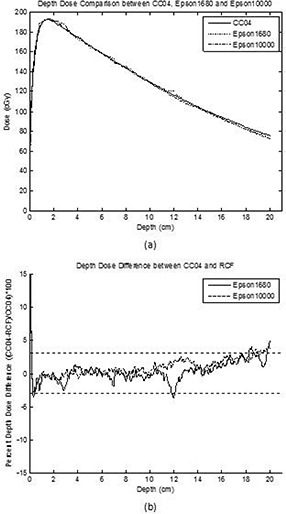
Percent depth dose for CC04, Epson 1680, and Epson 10000XL compared with CC04 (a); Percent dose difference between CC04 and both scanners (b). The dotted horizontal line is ± 3% error level.

Figure 4(a) shows the depth dose profiles for the CC04 ionization chamber, the Epson 1680 scanner, and the Epson 10000XL scanner. Figure 4(b) shows the depth dose difference between the CC04 ionization chamber and both scanners. From the surface down to 0.15 cm, the difference was large; however, with increasing depth, the difference declined to well within ± 3%. The mean of the difference from depth of 0.15 cm to 20 cm was 0.2%(1σ=1.4%) for the Epson 1680 scanner and 0.9%(1σ=1.2%) for the Epson 10000XL scanner. Table 5 presents the results for the percent depth doses for both scanners.

**Table 5:** Results for percent depth dose for the Epson 1680 and Epson 10000XL scanners.

	*All Data* {Epson1680/Epson10000XL}	*From depth of* 0.15cm{Epson1680/Epson10000XL}	*From depth of Dmax* {Epson1680/Epson10000XL}
Mean	0.4%/1.1%	0.2%/0.9%	0.3%/1.0%
Stand Deviation (1σ)	2.5%/2.5%	1.4%/1.2%	1.4%/1.2%

### D. IMRT results

Gamma index analysis with criteria of 3% dose difference and 3 mm DTA were used to evaluate 22 IMRT fields using the 2D diode array detector software. Planar doses from the treatment planning system were used as the reference. For the Epson 1680 scanner, 20 of the 22 IMRT fields had a passing rate greater than 90%. For the Epson 10000XL scanner, all 22 IMRT fields had a passing rate greater than 94%. The performance of RCFs was evaluated using the 2D diode array detector, which is a standard device for IMRT QA in our clinic, using the gamma index (Table 6). The dose from this device was also compared against the planar dose from the treatment planning system. For the most part, both scanners had passing rates greater than 95%. However, for the Epson 1680 scanner, 2 IMRT fields had a passing rate less than 90% (85.7%).

**Table 6:** Gamma index (3%/3 mm) results for all 22 IMRT fields for the Epson 1680 scanner, Epson 10000XL scanner, and MapCHECK 2D diode array detector.

*Field Number*	*Epson 1680*	*Epson 10000XL*	*MapCHECK*
1	99.5%	99.9%	100%
2	98.2%	98.1%	100%
3	95.8%	96.9%	98.2%
4	97.4%	97%	100%
5	99.2%	98.5%	99.4%
6	95.4%	96.8%	100%
7	98.8%	97.8%	98.6%
8	98%	99.3%	100%
9	98.8%	99.2%	100%
10	92.5%	96%	100%
11	98.7%	99.6%	99.4%
12	97.2%	97.2%	99.3%
13	93.3%	96.6%	97.3%
14	92.9%	95.9%	99%
15	92.5%	97.7%	95.1%
16	97.1%	98.8%	98.1%
17	96.5%	99.1%	97.4%
18	94.1%	97.3%	97%
19	90.6%	94.9%	95.7%
20	85.7%	94.5%	96.7%
21	85.7%	96.9%	97.4%
22	92.8%	96.2%	95%

## IV. DISCUSSION

The purpose of this study was to apply a methodology for which accurate absolute dosimetry can be done with RCF using a commercially available flatbed scanner without post‐scan correction. For this study, two different scanners were used, the Epson Expression 1680 and the Epson Expression 10000XL. Care must be taken during the irradiation and scanning process to minimize any inherent errors that may arise from either the film and/or the scanner used.

A significant error in performing absolute dosimetry using RCF can arise from the use of improper methods in generating dose calibration films. Every time a set of measurements is to be done, a new set of sensitometric curves should be generated. Dose calibration films (as described previously) have to be done in conjunction with the measurement film to properly characterize the conversion from OD to absolute dose. In order to do this correctly, the user should perform a quick output check on the linear accelerator to make sure that its daily output is within tolerances. Once this has been done, a known dose is delivered to each calibration film to generate a sensitometric curve. It is also important to keep in mind that the temperature and humidity level will affect the crystal polymerization process. Therefore, both calibration and measurement films should be handled together at all times. The orientation of the film during scanning will also affect pixel OD. For this reason, it is critical for the user to mark the films' orientation so that they can be consistently placed on the scanning bed. For this work, no film nonuniformity corrections were done. All films were placed in the center of the scanner bed to minimize scanner nonuniformity effects. Finally, only the red channel data from the TIFF files were used for analysis.^(15)^

Figures 2 and 3 show that the percent dose difference between RCF and CC04 for D50 and D80 was very favorable. Unfortunately, profiles beyond the 50% penumbra region showed much less agreement. One reason for this was that the dose beyond the 50% penumbra region was low (<10cGy). With such low doses, the percent dose difference analysis would show higher values at low doses. Another reason for such disagreement was the inherent scanner nonuniformity effect, which was not corrected for in this work. Because the profiles beyond the 50% penumbra region were larger than the $10\times 10\;{\text{cm}}^{2}$ field size, scanner nonuniformity had a noticeable effect. This was more apparent for the Epson 1680 scanner (Fig. 2(b) than for the Epson 10000XL scanner. As shown, profiles beyond 5 cm off‐axis showed more noticeable scanner nonuniformity for the Epson 1680 scanner than for the Epson 10000XL scanner. This finding is consistent with those reported by Lynch et al.^(9)^ They also reported different magnitudes of scanner nonuniformity for two scanners (up to 17% for the Epson 1680 scanner and up to 8% for the Microteck ScanMaker i900), which was similar to the effect observed in this study (i.e. a greater nonuniformity effect for the Epson 1680 scanner than for the Epson 10000XL scanner).

From observing the magnitudes of the scanner nonuniformity effect, we believe that when using the Epson 1680 scanner to scan a field size larger than $10\times 10\;{\text{cm}}^{2}$, a scanner nonuniformity correction may be appropriate. For the Epson 10000XL scanner, a scanner nonuniformity correction may be appropriate for field sizes larger than $15\times 15\;{\text{cm}}^{2}$. While a scanner nonuniformity correction may be necessary for these larger field sizes, they appear to be unnecessary for field sizes less than $10\times 10\;{\text{cm}}^{2}$ for the Epson 1680 scanner or less than $15\times 15\;{\text{cm}}^{2}$ for the Epson 10000XL scanner, both of which are consistent with most IMRT field sizes.

**Figure 5:**
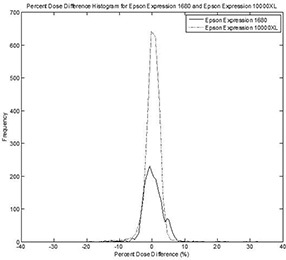
Mean of difference histogram for all flat and wedge fields (except the IMRT fields) using the Epson 1680 and Epson 10000XL scanners for D80. The solid line represents the Epson 1680 and the dotted line the Epson 10000XL.

Based on Table 7 and Fig. 5, it is safe to conclude that the method we have used has the ability to achieve an accuracy of 2%–4% when compared with CC04 profiles. This finding is consistent with those of others as well.^(10)^ ^,^ ^(11)^ Fiandra et al.^(10)^ reported an agreement of 3.6% between an Epson 1680 scanner and a 2D array Seven29 (T10024) model. Similarly, Paelinck et al.^(11)^ also reported an agreement of 2.5% between an Epson 1680 scanner and a diamond detector. The major difference between our study and these two studies is that we did not perform a significant scanner nonuniformity correction to account for the distinct bowing effect from the scanners. Thus, our approach is much more practical for clinical usage than the scanner nonuniformity correction methods employed by others, and it has good agreement with ionization chamber results.

**Table 7:** Mean of difference and absolute mean of difference for D80 for all flat and wedge fields for both scanners.

	*Epson 1680*	*Epson 10000XL*
Mean (1σ)	0.3% (2.9%)	0.4 (3.3%)
Absolute Mean (1σ)	2.2% (1.9%)	1.7% (2.8%)

The depth dose distribution from a depth of 0.15 cm to 20 cm for a field size of $10\times 10\;{\text{cm}}^{2}$ showed an agreement of 0.2%(1σ=1.4%) for the Epson 1680 scanner and of 0.9%(1σ=1.2%) for the Epson 10000XL scanner. This result is consistent with that of van Battum et al.^(12)^ who reported a depth dose agreement of ±0.6%. They measured depth dose profiles using a water tank with a CC04 ionization chamber and EBT film immersed in water during irradiation. The increase in depth dose difference at depths larger than 18 cm may be due to breakdown of charged particle equilibrium.

All 22 IMRT fields were taken from the treatment plans of four head and neck patients, with field dimensions ranging from 10 cm to 20 cm. The two fields that had a passing rate of 85.7% had both x‐ and y‐dimensions greater than 20 cm. Because of the large field size, these fields were susceptible to an over response by the Epson 1680 scanner due to scanner nonuniformity. Better passing rates were achieved by the Epson 10000XL scanner because of its larger lamp size and scanning area. It is worth mentioning that because the Epson 1680 scanner has a smaller scanning area than the Epson 10000XL scanner, scanning IMRT fields larger than $10\times 10\;{\text{cm}}^{2}$ will start to produce a noticeable effect from the scanner nonuniformity, which will adversely affect the passing rate. For such large field sizes, the user will observe that most failure points lie on the edges of the fields because of the scanner nonuniformity effect.

**Figure 6:**
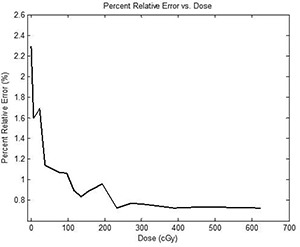
Plot of percent relative error versus dose for the Epson 10000XL scanner. At low doses (<100cGy), the percent relative error is greater than 1%, while at high doses (>100cGy), the percent relative error is less than 1%.

When delivering IMRT fields (doses ranging from ~35cGy to 150 cGy), all MU were increased by a factor of 5 (actual MU delivered to the films ranged from ~150MU to 525 MU) so that the doses delivered to the films were in a range where percent relative error was less than 1%. The percent relative error was obtained by irradiating small strips of RCF similar to the dose calibration films to known doses (0 cGy – 621 cGy). Means and standard deviations (1σ) of doses were obtained for all strips of films. The percent relative error was ascertained by taking the ratio between the standard deviation and the mean for a particular film strip. A small percent relative error (<1%) would indicate small OD variations. A large percent relative error (>1%) would indicate large OD variations. In other words, a small percent relative error would indicate a high level of precision by the RCF. From Fig. 6, it is evident that at low doses (<100cGy), the RCF have a percent relative error greater than 1%, while at higher doses (>100cGy), the EBT films have a percent relative error less than 1%. Overall, using RCF in conjunction with a commercially available flatbed scanner is appropriate for absolute dosimetry for IMRT. As indicated by the gamma index results for the scanners and 2D diode array detector, the detector had a higher passing rate than the film. This is partly due to the fact that the detector has much fewer evaluation points than the RCF, which has thousands of evaluation points. Also, unlike RCFs, the detector is not impacted by the scanner nonuniformity effect. In spite of these minor pitfalls, both scanners performed well relative to the detector in evaluating IMRT plans.

## V. CONCLUSIONS

The purpose of this work was to demonstrate accurate absolute dosimetry using GAFCHROMIC EBT film, in conjunction with a commercially available flatbed scanner, is feasible without the need for any scanner nonuniformity correction. In this work, the overall accuracy of flat and wedge profiles when compared with CC04 ionization chamber profiles was 2%–4% for both the Epson 1680 and the Epson 10000XL scanners. In general, IMRT fields do not exceed $15\times 15\;{\text{cm}}^{2}$. For fields that are smaller than or equal to $15\times 15\;{\text{cm}}^{2}$, these scanners' nonuniformities do not seem to have a significant effect on the gamma index evaluation when this method is used. On the other hand, for IMRT field sizes larger than $15\times 15\;{\text{cm}}^{2}$, the Epson 1680 scanner does have enough scanner nonuniformity effect to require a correction factor. However, the Epson 10000XL scanner does not require such a correction factor for these IMRT field sizes. For most IMRT fields, both scanners had an overall gamma index passing rate greater than 90%, except for two fields larger than $15\times 15\;{\text{cm}}^{2}$ when the Epson 1680 scanner was used. In conclusion, it is clear that a commercially available flatbed scanner can be used for accurate absolute dosimetry (agreement within 2%–4%) using RCF without significant scanner nonuniformity corrections.
